# The Toxicity Exerted by the Antibiotic Sulfadiazine on the Growth of Soil Bacterial Communities May Increase over Time

**DOI:** 10.3390/ijerph17238773

**Published:** 2020-11-26

**Authors:** Vanesa Santás-Miguel, Laura Rodríguez-González, Avelino Núñez-Delgado, Montserrat Díaz-Raviña, Manuel Arias-Estévez, David Fernández-Calviño

**Affiliations:** 1 Área de Edafoloxía e Química Agrícola, Facultade de Ciencias, Universidade de Vigo, 32004 Ourense, Galiza, Spain; vsantas@uvigo.es (V.S.-M.); laura.rodriguez.gonzalez@uvigo.es (L.R.-G.); mastevez@uvigo.es (M.A.-E.); 2 CITACA-Clúster de Investigación e Transferencia Agroalimentaria do Campus Auga, Universidade de Vigo, 32004 Ourense, Galiza, Spain; 3 Departamento de Edafoloxía e Química Agrícola, Escola Politécnica Superior de Enxeñaría, Universidade de Santiago de Compostela, 15705 Lugo, Galicia, Spain; avelino.nunez@usc.es; 4 Departamento de Bioquímica del Suelo, Instituto de Investigaciones Agrobiologicas de Galicia (IIAG/CSIC), 15705 Santiago de Compostela, Galicia, Spain; mdiazr@iiag.csic.es

**Keywords:** leucine incorporation, risk assessment, sulfonamide, veterinary, microbial community

## Abstract

The toxicity exerted by the antibiotic sulfadiazine on the growth of soil bacterial communities was studied in two agricultural soils for a period of 100 days. In the short-term (2 days of incubation), the effect of sulfadiazine on bacterial growth was low (no inhibition or inhibition <32% for a dose of 2000 mg·kg^−1^). However, sulfadiazine toxicity increased with time, achieving values of 40% inhibition, affecting bacterial growth in both soils after 100 days of incubation. These results, which were here observed for the first time for any antibiotic in soil samples, suggest that long-term experiments would be required for performing an adequate antibiotics risk assessment, as short-term experiments may underestimate toxicity effects.

## 1. Introduction

In recent decades, veterinary antibiotics have been widely used as therapeutic agents and even as promoters of animal growth in farming activities. The annual consumption of antibiotics in the European Union and in the USA was approximately 10,000 tons, with half of total consumption being for livestock production in the case of the European Union [1]. Between 30 and 80% of the veterinary antibiotics administered to animals are excreted in feces and urine as unaltered forms, reaching the terrestrial environmental due to repeated spreading of manure/slurry in agricultural lands [2]. Therefore, high amounts of antibiotics are spread on agricultural soils each year [3]. Among veterinary antibiotics, the group of sulfonamides is one of most important in relation to its use in pig production, and specifically in the European Union [4]. Moreover, sulfonamide antibiotics show a high persistence in manures and soils [5,6,7], and are considered among those that present a higher risk regarding vulnerability of EU soils [8]. Among them, sulfadiazine (4-amino-N-(2-pyrimidinyl) benzene sulfonamide) is one of the most frequently used in veterinary medicine, especially in pigs [9]. Sulfadiazine (SDZ) is a broad-spectrum antibiotic with effect on many bacterial groups [10,11,12], because it is a competitive inhibitor of the bacterial enzyme dihydropteroate synthetase, and therefore of folic acid synthesis. Therefore, once in the soil it may affect to many no-target bacteria, with undesirable side-effects of reducing soil and environmental quality. Thus, the presence of sulfadiazine in soils may decrease N mineralization [13], and soil microbes’ enzymatic activity [14], as well as increase bacterial community tolerance to sulfadiazine [15], or increase the presence of antibiotic resistance genes in water bodies [16,17].

Assessments focusing on decreasing risks (risk mitigation) are increasingly important in order to protect the biodiversity and ecological functioning of ecosystems. Since it is not easy to predict the relationship between the chemical structure of organic compounds and their effect on soil microbial communities [18], a broad range of toxicological tests should be conducted to determine the toxicity of different pollutants onto soil microorganisms. However, SDZ remains as an understudied antibiotic in relation to its implications in the eventual effects of soil pollution on microbial communities [19,20]. In addition, most of the available results are based on methods focusing on the quantification of parameters/properties such as soil respiration, enzymatic activities, or microbial community structure [21], which are techniques with clearly lesser sensitive potential to detect toxicity than bacterial growth [22,23]. Further, bacterial community growth rates have also been shown to be sensitive measurements to detect changes in other environmental conditions, like pH and heavy metal contamination [24,25]. Therefore, the effect of sulfadiazine on the growth of bacterial communities should be analyzed to study the effects of this antibiotic with a sensitive end-point, in order to provide evidence to enable management practices that can protect the soil ecosystem.

In addition to the direct effects of organic compounds on soil microbial communities, the persistence of these effects in the environment is also of importance. Organic compounds are subjected to physical, chemical, and biochemical processes in the soil, such as adsorption, degradation, and transformation [26], and hence their effects on soil microorganisms may change with time. The effect of time on toxicity can differ depending on the type of toxic compound, soil characteristics, or the toxicity test employed. Usually, toxicity will decrease with time due to dissipation of the compound [23,27,28,29]. However, an increased toxicity with time is also possible, as suggested for the herbicide mecoprop using soil respiration as toxicity test in an acid soil [30], and recently confirmed for terbutryn [31].

Taking all these facts into consideration, the aim of the present work was to evaluate the effect of the antibiotic SDZ on the growth of soil bacterial communities both short-term (direct toxicity) and over time. Two acid soils were used for the toxicity tests, and the ^3^H leucine incorporation method was employed for the estimation of bacterial community growth in those soils. Therefore, this is the first study focusing on the effect of time on the toxicity exerted by the antibiotic sulfadiazine on bacterial community growth. The main objective of this research was to provide novel knowledge regarding risk assessment related to accumulation of antibiotics in agricultural soils.

## 2. Material and Methods

### 2.1. Experimental Design

In order to check the temporal effect of sulfadiazine on the growth of soil bacterial communities, two soil samples differing in their total carbon content were selected. For each of the two soils, aliquots of 252 g (dry weight) were placed into 500-mL polypropylene jars and subsequently rewetted up to 60–80% of the water holding capacity, and incubated for 1 week at 22 °C in the dark. This incubation time was sufficient for the recovery of microbial activity of a dry soil after rewetting [32]. After microbial recovery, each soil was distributed in 36 polypropylene jars of 50 mL (7 g of soil into each jar), resulting in a total of 72 microcosms (36 per soil). Subsequently, different amounts of SDZ were added to the soil microcosms, reaching 12 different concentrations of SDZ (0, 0.002, 0.01, 0.03, 0.12, 0.5, 2.0, 7.8, 31.3, 125, 500, and 2000 mg·kg^−1^) per soil, which was carried out per triplicate. Sulfadiazine was added to the soil microcosms using talc powder as a carrier, in order to equalize the amount of dry material added to each microcosm, thereby facilitating SDZ mixing with soil [33]. Then, the bottles were sealed and incubated for 100 days at 22 °C in the dark. During this period, bacterial community growth was estimated on each microcosm after 2, 4, 8, 16, 32, 64, and 100 days of incubation. The soil microcosms were aerated at the time of sampling by removing the lid for 30 min, while the soil moisture was maintained by adding water when needed.

### 2.2. Materials

Sulfadiazine (CAS 68-35-9; ≥99.0% purity) was supplied by Sigma–Aldrich (Steinheim, Germany), and was used for performing the assessment of SDZ toxicity on the bacterial community in soil samples. Talc (CAS 14807-96-6) was supplied by Sigma–Aldrich (Steinheim, Germany).

The two selected soil samples were previously analyzed by Conde–Cid et al. [34]. They were sampled in A Limia, an agricultural area located in the southeast of Galicia (NW Iberian Peninsula). The soil samples were taken with an Edelman probe, at a depth of 0–20 cm, along two plots, sampling a total of 10 subsamples per plot (total amount of 2 kg of soil per sample). The subsamples collected in each plot were mixed into a single composite sample for each soil. Subsequently, once in the laboratory, the composite soil samples were air-dried, sieved through a 2-mm mesh, homogenized, and stored in polypropylene bottles until analysis.

### 2.3. Characterization of Soils

The proportions of sand (particle size 2–0.05 mm), silt (0.05–0.002 mm), and clay (<0.002 mm) of the soils were determined by wet sieving for the size fractions greater than 0.05 mm, and using the international pipette method for those smaller [35]. Soil pH was determined in water (soil ratio: 1:2.5), using a combined glass electrode [35]. Total carbon and total nitrogen were determined by elemental analysis in a LECO CHN-1000 (LECO Corporation, St. Joseph, MI, USA). Exchangeable basic cations (Ca, Mg, Na, and K) were extracted with 0.2 M NH_4_Cl [36], while exchangeable Al was extracted with 1 M KCl [37], and then determined by flame atomic absorption (Ca, Mg, and Al) or emission spectroscopy (Na and K). The effective cation exchange capacity (eCEC) was estimated as the sum of the exchangeable basic cations and Al. Available phosphorus was extracted using 0.5 M NaHCO_3_ and determined using the phosphomolybdic complex method [38].

Table 1 shows the main characteristics of the studied soils. Briefly, the soils were sandy loam (soil 1) and sandy clay loam (soil 2) in texture, and presented total carbon and nitrogen contents of 1.07 and 0.09% (soil 1), and 3.1 and 0.25% (soil 2). The pH values in water were 4.8 (soil 1) and 4.6 (soil 2), and eCEC values were 4.1 for soil 1, and 5.3 cmol_c_ kg^−1^ for soil 2. The bioavailable phosphorus was 225 for soil 1 and 169 mg·kg^−1^ for soil 2.

### 2.4. Estimation of Bacterial Community Growth

Bacterial community growth was estimated extracting the bacterial community from all soil microcosms, using homogenization/centrifugation and leucine (Leu) incorporation into bacteria [39], in accordance with the method of Bååth et al. [40]. The method determined the bacterial protein synthesis, which was used as a proxy for bacterial growth, by estimating ^3^H-Leu incorporation into extracted bacteria. Briefly, 1 g of soil (fresh weight) was mixed with 10 mL of distilled water using a multi-vortex shaker for 3 min, at maximum intensity, followed by low-speed centrifugation at 1000× *g* for 10 min, creating a bacterial suspension in the supernatant. Then, an aliquot of this bacterial suspension (1.5 mL) was transferred to a 2-mL micro-centrifugation tube, and 2 µL of [^3^H]Leu (3.7 MBq mL^−1^ and 0.574 TBq mmol^−1^; Perkin Elmer, Waltham, MA, USA) was added together with non-labeled Leu to each tube, resulting in 275-nM Leu in the bacterial suspensions. Then, the micro-tubes were incubated for 2 h at 22 °C in the dark, and the growth stopped with 75 µL of 100% trichloroacetic acid after the incubation period. Later, the bacteria in the tubes were washed as described by Bååth et al. [40]. Finally, ^3^H radioactivity was determined using scintillation liquid counting (Tri-Carb 2810 TR, Perkin Elmer, Waltham, MA, USA).

### 2.5. Data Analysis and Statistics

Data corresponding to the bacterial growth estimated as a function of SDZ concentration were normalized respect to the control (sample without antibiotic) for each soil, and plotted (as relative bacterial growth vs. log antibiotic concentration) using results obtained for each soil mixture and the antibiotic.

The statistical significance of differences between the control soil and soil samples spiked with the various SDZ concentrations was estimated using one-way ANOVA and Dunnet’s post hoc test (*p* < 0.05). The statistical analyses were performed using IBM SPSS statistics 23.0 and JMP Pro 13.0 for Mac (SAS Institute Inc., Cary, NC, USA).

## 3. Results and Discussion

**Figure 1:**
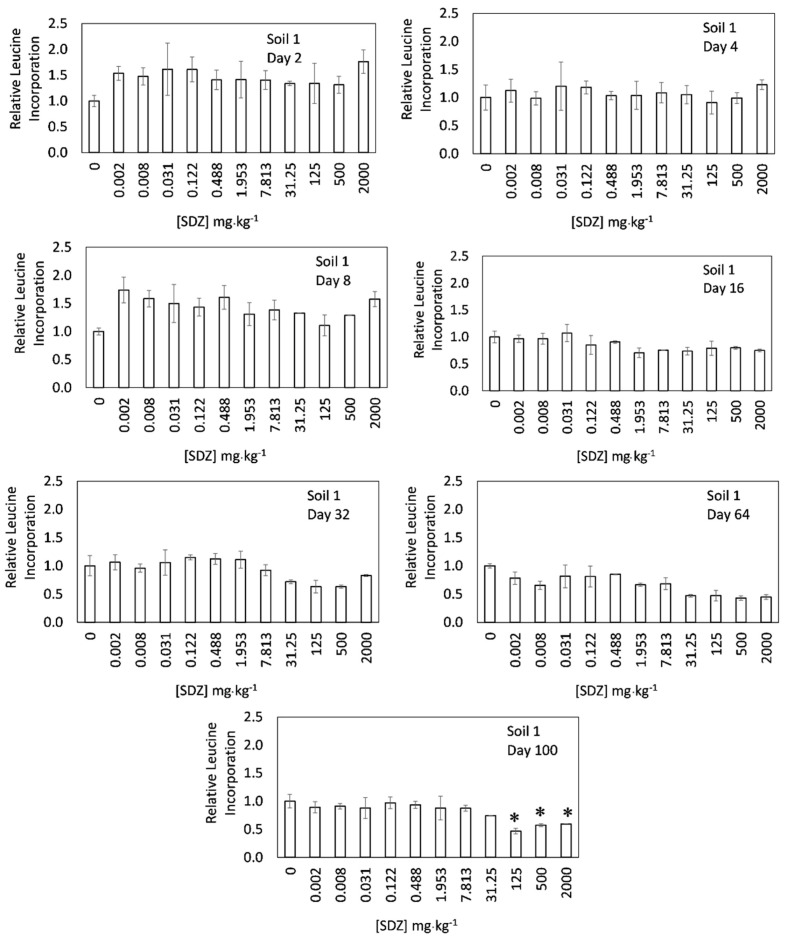
Relative bacterial community growth (estimated as ^3^H Leucine incorporation) in soil 1 as a function of sulfadiazine concentration added for different incubation times. Error bars show the standard errors from average values (*n* = 3), and * indicates significant differences with respect to the control (*p* < 0.05).

**Figure 2:**
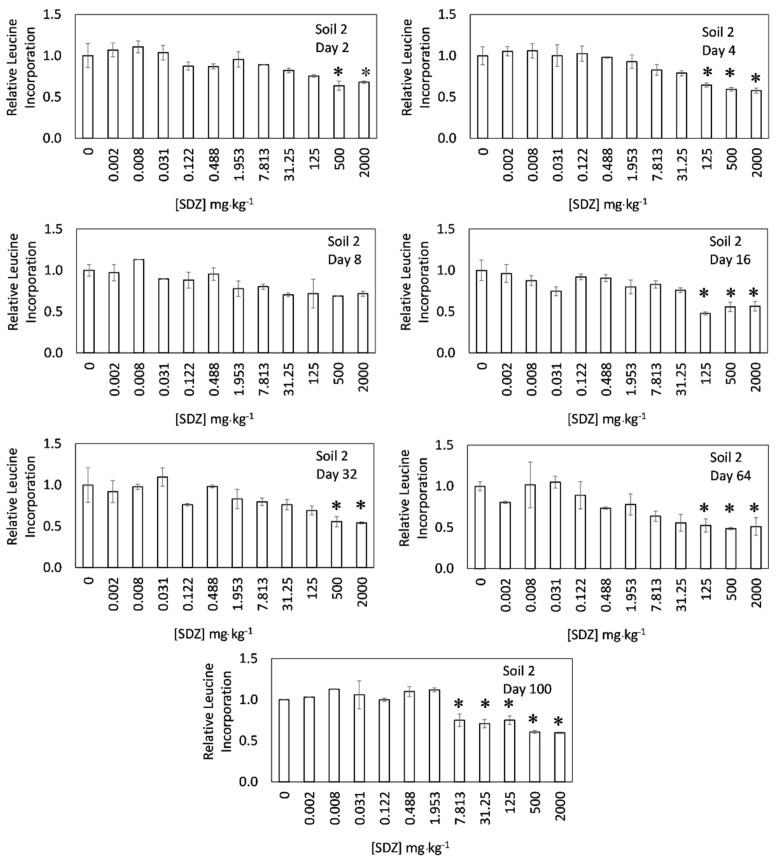
Relative bacterial community growth (estimated as ^3^H Leucine incorporation) in soil 2 as a function of sulfadiazine concentration for different incubation times. Error bars show the standard errors from average values (*n* = 3), and * indicates significant differences with respect to the control (*p* < 0.05).

Figure 1 and Figure 2 show the relative growth of soil bacterial communities in response to increasing concentrations of sulfadiazine (0–2000 mg·kg^−1^) for different times of incubation (2–100 days), i.e., the figures show the effect of time on the toxicity exerted by sulfadiazine on the growth of bacterial communities. In the short-term, after two days of incubation, the addition of SDZ to soil 1 (Figure 1) did not affect negatively the growth of bacterial communities for any of the concentrations tested (up to 2000 mg·kg^−1^). In fact, the bacterial community growth even increased for most of the concentrations used, although those increases were not statistically significant. However, in soil 2 (Figure 2), the bacterial community growth decreased for the higher SDZ doses, achieving significant reductions in relation to the control for the doses of 500 and 2000 mg·kg^−1^. However, the magnitude of decrease for the highest SDZ dose (2000 mg·kg^−1^) was relatively low (32%), if compared with results previously reported for other antibiotics, such as tylosin [33,41], tetracyclines [22,28,29,33], or streptomycin [22]. It is worth noting that, despite the low adsorption previously reported for SDZ onto soils [42], the short-term effect of SDZ on bacterial community growth was relatively low. This unmarked toxicity may be attributed to the scarce solubility of SDZ [6,43,44]. Demoling et al. [27] found a reduction of bacterial community growth reaching around 55% in a soil amended with 500 mg·kg^−1^ of another sulfonamide antibiotic (sulfamethoxazole), which meant a higher toxicity than that found for SDZ in the present work. The difference may be also attributed to their variated solubility values, since that of sulfamethoxazole (610 mg L^−1^) was one order of magnitude higher than SDZ solubility (77 mg L^−1^) [45]. In other works, using different endpoints, a low direct effect on microbial communities was also found for SDZ. Thus, in an experiment using 50 mg·kg^−1^ of SDZ, no effect was found on respiration activity or bacterial community structure [19]. In another work, using PLFAs (Phospholipid Fatty Acids), Hammesfahr et al. [20] found an increase in microbial biomass (total PLFAs) when the soil was polluted with 100 mg·kg^−1^ of the antibiotic, but a modification in the microbial community structure was also reported. Overall, the short-term results found in the present work, together with results from previous works, suggest that SDZ accumulation in soils causes low negative direct effects on soil microbial communities.

Figure 1 also shows a clear increase of SDZ toxicity over time in the experiments performed with soil 1. For various of the incubation times, the increases in toxicity were not significant, but after 100 days of incubation, the increase in toxicity became significant for SDZ concentrations ≥125 mg·kg^−1^. For soil 2, SDZ toxicity also increased with time, achieving significant decreases in the bacterial community growth after 100 days of incubation when SDZ concentrations were ≥7.8 mg·kg^−1^. These results showed that SDZ toxicity may be not only persistent with time, but also may increase, i.e., using only short-term experiments, the research may underestimate the toxicity of this antibiotic. These results can be considered somehow surprising, since organic compounds (including antibiotics) generally decrease their toxicity with time. As examples, decreases in toxicity of organic compounds were previously found for atrazine when examining effects on microbial biomass [46], as well as for fomesafen regarding effects on urease activity [47], or tetracycline regarding its effect on bacterial community tolerance [28,29]. This decrease is reasonable, since the concentrations of these organic compounds were reduced with time due to degradation [48], or suffered immobilization in the soil via ageing processes [49], or microbial communities developed tolerance to the organic compound [50]. For SDZ, Hammesfahr et al. [20] found that the concentration of the antibiotic in soils highly decreased with time, i.e., its half-life in soils was between 5.6–8.5 days for a SDZ concentration of 100 mg·kg^−1^. Therefore, a decrease in toxicity was expected. However, in the soils of the present study the toxicity was significant for a similar SDZ concentration (125 mg·kg^−1^, or even lower) after 100 days of incubation. Therefore, the persistence/increase of toxicity initially caused by SDZ with time may continue in the soil even when the antibiotic has disappeared from that environment.

The increases of toxicity with time for organic compounds are less common, but were recently described for the herbicide terbutryn [31]. However, to our knowledge, it is the first time that the increase of toxicity on soil microbial communities with time has been described for a veterinary antibiotic. We hypothesized two possible mechanisms for this increase in toxicity, although it would need further future studies. One possibility is that some compounds resulting from SDZ degradation became more toxic than SDZ itself, similar to the case described for carbendazim, which is a metabolite derived from the fungicide benomyl [51]. As SDZ was progressively degraded, the concentration of degradation products increased, with a subsequent possible rise in toxicity with time. Two major metabolites of SDZ are N4-acetylsulfadiazine and 4-hydroxysulfadiazine [52], but their toxicity on soil microbial communities has not yet been studied. Another possibility could be a chronic effect of SDZ and its metabolites on microbial growth, due long-term microbial stress. In this hypothesis, SDZ and its metabolites would have little or no effects in the short-term, but negative effects would appear with time, due to high metabolic requirements affecting to the microorganisms when fighting against the toxicants present in that medium [53]. More future research would be needed to clarify the mechanism behind the increase/persistence of SDZ toxicity with time of exposure.

The results found in the present work have important implications for the ecotoxicological assessment of antibiotic effects on bacterial communities and risk assessment analyses (ERA). Current ERA protocols [54,55] assume that the impacts of toxicants will decrease with incubation time [56], and therefore, in the case of sulfadiazine, they may underestimate the risks associated with the accumulation of this antibiotic in soils. In view of that, it is important to reconsider the duration of current standardized ecotoxicological tests in order to propose longer periods of incubation. Time extensions, together with the use of more sensitive endpoints, may contribute to improve ERA analyses and overcome the recent criticisms received by current methods due to overlooking important effects of toxicant on soil microbiota [57].

## 4. Conclusions

In the present work, using short incubation times to check SDZ toxicity would result in a conclusion indicating the absence of negative effects of SDZ on soil bacterial growth, even at concentrations as high as 2000 mg·kg^−1^ for one of the soils tested (soil 1). However, a relevant finding of this study is that, for long incubation times (100 days), toxicity effects could be detected even for concentrations lower than 10 mg·kg^−1^ of SDZ. Taking into account that most studies regarding toxic effects on soil microorganisms are performed in the short- or medium-term, they may underestimate overall toxicity that could affect to soil microorganisms. Therefore, it is suggested that, for potentially toxic substances, risk assessment methodologies and procedures should be adapted to take into consideration the possible existence of long-term effects, even in cases of absence of short-term effects. It could be of relevance for studies in relation to both environment aspects and public health. These findings are of special importance for improving the current procedures dealing with antibiotics’ risk assessment in soils, suggesting the need for new methodologies that take into account potential delayed negative effects of antibiotics on soil microbes. Therefore, further research is needed to finally propose appropriate changes that could improve the current methodologies.

## Figures and Tables

**Table 1:** General characteristics of the soils used.

Parameter	Soil	
	1	2
Sand (%)	70 ± 2	65 ± 2
Silt (%)	12 ± 1	13 ± 1
Clay (%)	18 ± 1	21 ± 2
Texture	Sandy loam	Sandy clay loam
pH_w_	4.8 ± 0.1	4.6 ± 0.1
C (%)	1.1 ± 0.1	3.1 ± 0.2
N (%)	0.09 ± 0.01	0.25 ± 0.04
Ca_e_ (cmol_c_ kg^−1^)	1.53 ± 0.06	1.51 ± 0.21
Mg_e_ (cmol_c_ kg^−1^)	0.41 ± 0.03	0.52 ± 0.02
Na_e_ (cmol_c_ kg^−1^)	0.25 ± 0.03	0.21 ± 0.01
K_e_ (cmol_c_ kg^−1^)	1.27 ± 0.14	0.93 ± 0.09
Al_e_ (cmol_c_ kg^−1^)	0.61 ± 0.05	2.16 ± 0.07
eCEC (cmol_c_ kg^−1^)	4.08 ± 0.31	5.33 ± 0.40
P_available_ (mg·kg^−1^)	225 ± 34	169 ± 9	

pH_W_ is pH measured in water; C is total carbon; N is total nitrogen; eCEC is the effective cation exchange capacity (cmol_c_ kg^−1^); X_e_ is exchangeable concentration of the element.
